# Supplementary material for: Targeting Tumor Microenvironment Interactions in Chronic Lymphocytic Leukemia Using Leukotriene Inhibitors
Source: Int J Mol Sci. 2025 Feb 28;26(5):2209. doi: 10.3390/ijms26052209 (PMC11899779; doi:10.3390/ijms26052209)
Supplement: Supplementary file 1 [file ijms-26-02209-s001.zip › ijms-3458977-supplementary.pdf]

International Journal of *Molecular Sciences*

Targeting Tumor Microenvironment Interactions in Chronic Lymphocytic Leukemia Using Leukotriene Inhibitors

Laia Sadeghi [1,*], Magali Merrien [2], Magnus Björkholm [3], Anders Österborg [4,5], Birgitta Sander [2], Hans-Erik Claesson [3] and Anthony P. H. Wright [1]

[1] Department of Laboratory Medicine, Division of Biomolecular and Cellular Medicine, Karolinska Institutet, 17177 Stockholm, Sweden; anthony.wright@ki.se
[2] Department of Laboratory Medicine, Division of Pathology, Karolinska Institutet, 17177 Stockholm, Sweden; magali.merrien@ki.se (M.M.); birgitta.sander@ki.se (B.S.)
[3] Department of Medicine Solna, Karolinska Institutet, 17177 Stockholm, Sweden; magnus.bjorkholm@ki.se (M.B.); hans-erik.claesson@ki.se (H.-E.C.)
[4] Department of Oncology-Pathology, Karolinska Institutet, 17176 Stockholm, Sweden; anders.osterborg@ki.se
[5] Department of Hematology, Karolinska University Hospital, 17176 Stockholm, Sweden

* Correspondence: laia.sadeghi@ki.se

Figure S1: Primary CLL cells from different patients migrate and adhere differently to stromal cell

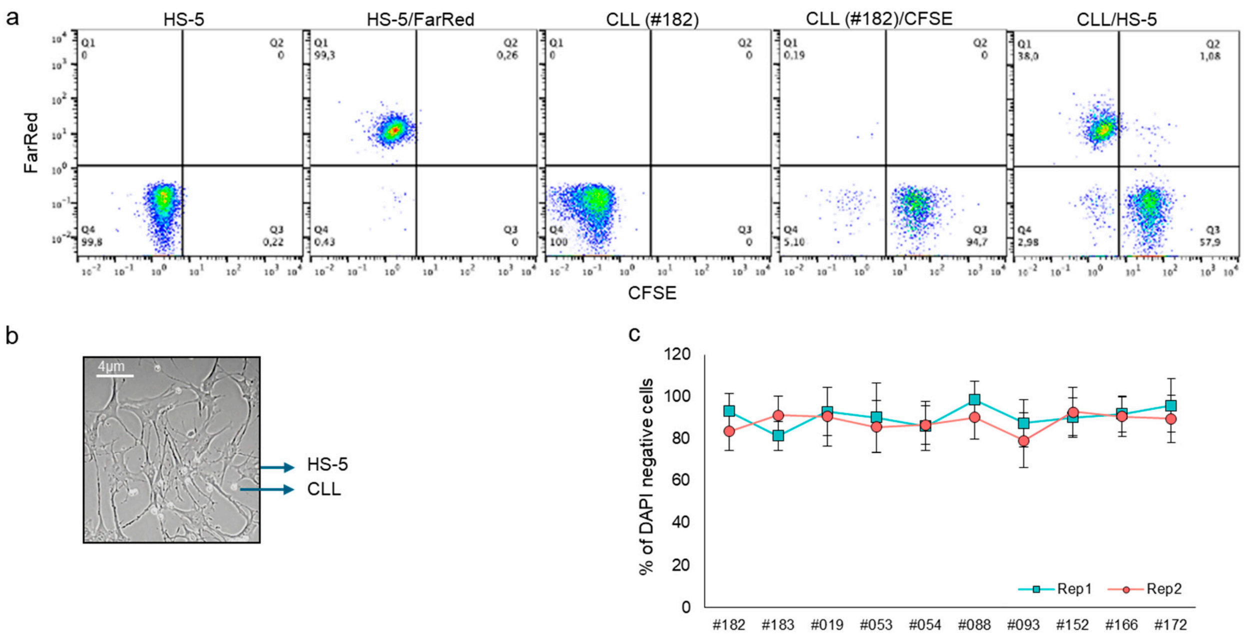

(a) Flow cytometry gating strategy used to separate primary CLL cells from HS-5 stromal cells in co-culture. CLL cells were labeled with CFSE (Carboxyfluorescein succinimidyl ester), and HS-5 stromal cells were labeled with Far-Red dye. The two cell populations were distinguished based on their fluorescence signals.

(b) Bright-field microscopy image of a co-culture of primary CLL cells with HS-5 stromal cells after 4 hours, captured using a 40× objective lens.

(c) Suspension fractions of CLL cells in co-culture were stained with DAPI, and the number of DAPI-negative cells was calculated as a percentage of total events in each well. Error bars represent variability among four wells used for sample in each technical replicate.

Figure S2: MK886 and zileuton dose response curve

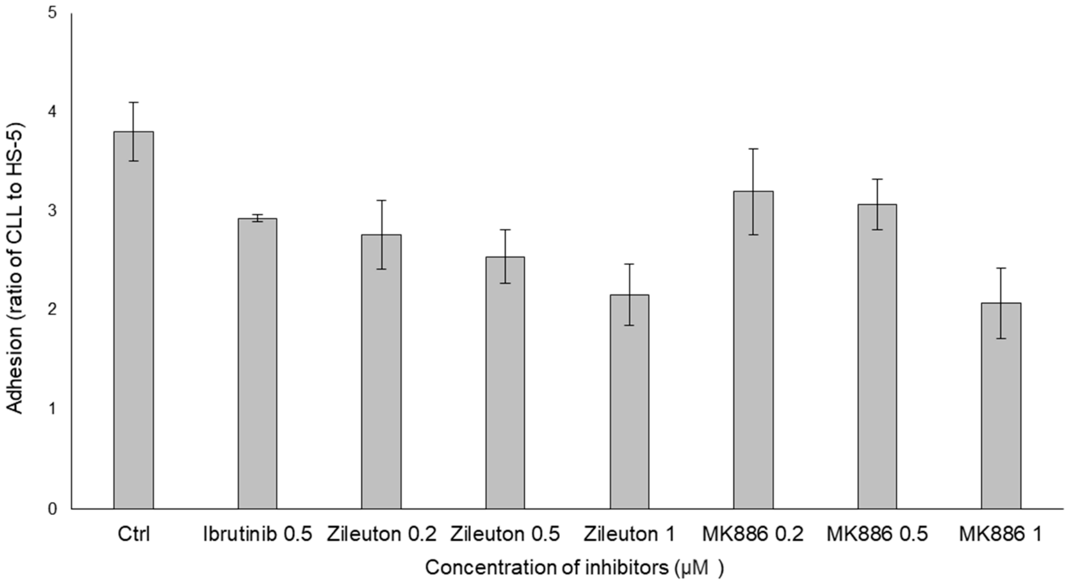

Assessing the optimal concentration of inhibitors for maximum reduction of CLL adhesion to stromal cells. Primary CLL cells were co-cultured with stromal cells for 4 hours in the presence of 0.5 µM ibrutinib and varying concentrations of MK886 and zileuton (0.2, 0.5, and 1 µM). The number of adherent cells was quantified using flow cytometry.

Supplementary Table S1: This table presents the results of the Kruskal-Wallis test to assess the association between different genetic mutations and the effects of various single-molecule inhibitors on primary cell adhesion. The p-values indicate whether there are statistically significant differences in adhesion based on mutation status across different inhibitors.

**Mutation**	**Adhesion**	**Ibrutinib**	**MK886**	**Zileuton**
IGHV mutation	0.04	0.03	0.09	0.04
del (17p)	0.03	0.09	0.05	0.08
del (13q;14)	0.04	0.05	0.06	0.02
del (11q)	0.08	0.07	0.08	0.03
Trisomi 12	0.08	0.09	0.11	0.06
